# Blockchain Security and Privacy for the Internet of Things

**DOI:** 10.3390/s21030892

**Published:** 2021-01-28

**Authors:** Marco Picone, Simone Cirani, Luca Veltri

**Affiliations:** 1 Department of Sciences and Methods for Engineering (DISMI), University of Modena and Reggio Emilia, Via Amendola 2, Pad. Morselli, 42121 Reggio Emilia, Italy; marco.picone@unimore.it; 2 Director of Engineering, Caligoo srl, Via Don Minzoni, 112, 43024 Taneto di Gattatico (RE), Italy; simone.cirani@unipr.it; 3 Department of Engineering and Architecture, University of Parma, Parco Area delle Scienze, 181/A, 43124 Parma, Italy

The Internet of Things (IoT) is of continuously growing interest for research and industry. IoT technologies are reaching maturity, as demonstrated by the increasing number of IoT applications in several markets ranging from smart homes to smart factories and Industry 4.0, with the so-called Industrial Internet of Things (IIoT).

However, some issues remain despite this success. Amongst them, the main issue that may slow down the adoption of IoT relates to security. The heterogeneity in terms of protocols, operating systems, and devices, combined with poor adoption of standard solutions, create insecure designs, architectures, and deployments. Furthermore, IoT applications are often associated with sensitive data, core infrastructures, and assets, thus making them attractive in terms of vulnerability, data breaches, and denial of service attacks.

Unfortunately, conventional security approaches tend to be inapplicable in the IoT due to the limitations of the resources of IoT devices and the decentralized nature of IoT architectures.

Blockchain is a technology that is currently receiving great attention and may help in providing security in IoT scenarios. The decentralized architecture of blockchains, together with the ability to provide data immutability and non-repudiation services, seem to make blockchain a promising technique for securing IoT and protecting user/data privacy.

The Special Issue “Blockchain Security and Privacy for the Internet of Things” seeks to explore the innovative developments, technologies, and challenges related to blockchain, security, and privacy for the IoT coming from both the latest research activities and ongoing projects. The presented topic is characterized by many open challenges that need to be solved or improved, and for this purpose, while several manuscripts have been received, only 15 original and high-quality manuscripts were selected for this Special Issue. Each manuscript was reviewed by several reviewers and went through multiple rounds of the peer-review process.

We had two interesting review papers [1,2] presenting, respectively, the current evaluation of blockchain technologies and their applicability to eHealth privacy management. In particular, the first aims at providing a systematic review of current blockchain evaluation approaches and identifying the corresponding challenges and limitations towards their utilization. The authors outline the main metrics related to the blockchain evaluation and propose an appealing modeling and analysis classification based on a critical literature review while also identifying the current open challenges as future perspectives and innovations. The latter paper instead presents the state of the art of decentralized identity management using blockchain to highlight possible opportunities for adopting decentralized identity management approaches for future health identity systems.

The authors of [3] present an IoT adaptive dynamic blockchain networking method based on discrete heartbeat signals. The core aspect of the proposed method is to set a different monitoring time for each group of nodes acting as discrete heartbeat monitoring signals. When the number of nodes gradually decreases, the network can dynamically adapt and react to this process, even when more than 1/3 of the IoT nodes are offline. The method also has the advantage of a short network expectation recovery time, able to avoid instantaneous system blocks due to the thundering herd effect. 

In [4], the authors propose a secure and lightweight fine-grained data sharing scheme for a mobile cloud computing scenario. The aim was to outsource the majority of time-consuming operations from resource-constrained mobile devices to the cloud. The introduced novelty is associated with the possibility: (i) To support verifiable outsourced decryption, i.e., the mobile user can ensure the validity of the transformed ciphertext returned from the cloud server, (ii) to outsource decryption for intensive computing tasks during the decryption phase without revealing user data or decryption key, and (iii) to achieve a CCA security level. The concrete security proof and performance analysis illustrates how the novel scheme is secure and suitable for mobile cloud computing environments.

In the context of Smart City application and use cases, [5] presents a blockchain-based and distributed Security Information and Event Management (SIEM) system. The proposed SIEM relies on blockchain technology to securely store and access security events associated with IoT sentinels that are in charge of shielding groups of distributed and connected devices. The IoT sentinels can be deployed within several smart city assets, such as smart hospitals, smart transport systems, and smart airports, among others, ensuring a satisfactory level of protection. The blockchain guarantees the non-repudiation and traceability of the registry of security events due to its features. The authors demonstrate the feasibility of the proposed approach through an extended evaluation and implementation based on Ethereum and validated through different use cases and experiments.

The authors of [6] propose a hybrid model based on recurrent neural networks (RNN) in the context of secure IoT–blockchain data for Industry 4.0 in the food sector. The authors adopt advanced deep learning (ADL) techniques, long short-term memory (LSTM), and gated recurrent units (GRU) as a prediction model, together with a genetic algorithm (GA) in order to optimize the parameters of the hybrid model. They select the optimal training parameters by means of GA and finally cascade LSTM with GRU. The aim of the manuscript was to help supply chain practitioners take advantage of the state-of-the-art technologies and to also help the industry make policies according to the predictions of ADL. 

In [7], an architectural framework for IIoT is proposed in order to provide authentication and guarantee integrity. The illustrated approach addresses the security by design principle while combining some of the emerging technologies like Secure Multi-Party Computation (SMPC) for grounded policy rules and Distributed Ledger Technology (DLT) for an immutable and transparent registry.

In the challenging ecosystem of intelligent mobility and transportation systems, the authors in [8] present a blockchain-based architecture as a trust reference infrastructure to protect user privacy and provide trustworthy services to users. It is also compatible with the legacy intelligent transportation system (ITS) infrastructure and services. In addition, the hierarchical organization of chains enables the scalability of the system, while the use of smart contracts provides a flexible way for introducing new services in the ITS. The proposed architecture is demonstrated by a proof of concept implementation based on Ethereum, and the illustrated test results show the feasibility of the proposed architecture.

In [9], the authors propose a novel blockchain-based platform for monitoring patient vital signs using smart contracts. The proposed system is designed and developed using hyperledger fabric, which is an enterprise-distributed ledger framework for developing blockchain-based applications. The presented approach provides several benefits to the patients, such as an extensive, immutable history log and global access to medical information from anywhere at any time. The Libelium eHealth toolkit is used to acquire physiological data, and the performance has been evaluated in terms of transaction per second, transaction latency, and resource utilization using a standard benchmark tool known as Hyperledger Caliper, showing how the proposed system outperforms the traditional health care system for monitoring patient data.

In [10], a decentralized and trustworthy Capability-Based Access Control scheme relying on the Ethereum smart contract technology is proposed. In this scheme, targeting the IoT context, a smart contract is created for each object in order to store and manage the capability tokens (i.e., data structures recording granted access rights) assigned to the related subjects and to verify the ownership and validity of the tokens for access control. The presented novel management solution achieves more fine-grained and flexible capability delegation while also ensuring the consistency between the delegation information and the information stored in the tokens. The solution has been implemented through a locally constructed Ethereum blockchain network to demonstrate its feasibility and to measure the monetary cost of the scheme in terms of gas consumption, and compare the scheme with existing schemes proposed by other researchers. 

Paper [11] investigates the issues associated with the use of heterogeneous devices and the runtime verification of task fulfillment with different constraints in real-world IoT scenarios. The proposed solution delegates the responsibility of a verification monitor from a centralized architecture to a decentralized one using blockchain technology. They present a smart contract-based task management scheme to provide runtime verification of device behaviors and to allow trustworthy access control to these devices. The business logic of the proposed system is specified by the smart contract, which automates all time-consuming processes cryptographically and correctly. A comprehensive evaluation experiment has been conducted, and the reported results indicate the effectiveness and efficiency of the proposed approach.

An IoT security transmission and storage solution regarding sensing images for blockchain is proposed by the authors in [12]. The proposed solution intelligently senses user image information and divides the sensed data into intelligent blocks. Different blocks of data are encrypted and transmitted securely through intelligent encryption algorithms. In the end, signature verification and storage are performed through an intelligent verification algorithm. Compared with the traditional IoT data transmission and centralized storage solutions, the introduced approach allows for a combination of the IoT with blockchain, exploiting the advantages of blockchain decentralization, high reliability, and low cost to transfer and store users’ image information securely. Security analysis proves the solidity of the solution and how it can ensure the security of user image information during transmission and storage. 

In the Smart Grid application scenario, the authors of [13] introduce a blockchain architecture based on the use of sidechains in order to make the system scalable and adaptable. The authors adopted three blockchains to ensure privacy, security, and trust in the overall system. Furthermore, in order to universalize the proposed solution, they introduced the Open Smart Grid Protocol and smart contracts. Illustrated results show how security and privacy are guaranteed through the proposed architecture, making it feasible for implementation in real systems and deployments.

The authors in [14] propose a blockchain-based trust management system with a lightweight consensus algorithm with the aim to provide a distributed trust framework for routing nodes in mobile ad-hoc networks (MANETs). The blockchain addresses most of the security issues in the optimized link-state routing protocol, in which every node is performing the security operation individually and in a repetitive manner. Nevertheless, using predefined principles, the routing nodes in the proposed scheme can collaborate to defend themselves from attackers in the network. The experimental results show how the proposed consensus algorithm is suitable for use in the resource-hungry MANET with reduced validation time and less overhead. Furthermore, the attack detection overhead and time also decrease while providing a scalable and distributed trust among the routing nodes. 

Paper [15] presents a novel authentication algorithm to manage the insiders on the cloud through a blockchain-based authentication mechanism. The proposed approach introduces the following contributions: The proposed mechanism authenticates both insider and outsider actors, and the peer-to-peer authentication is provided to the cloud database user via a blockchain mechanism. The proposed solution has been tested using a Scyther formal system tool against various attacks to evaluate the performance. The presented results showed how the system is highly efficient and successful in mitigating various outsider and insider threats and can also enhance the security of the cloud environment by identifying different possible attacks.

Finally, we would like to thank all authors and reviewers contributing to this Special Issue, the former for their original solutions and the latter for improvement suggestions. Their excellent work has allowed us to present novel and interesting contributions in the field IoT and blockchain technologies.
